# Competence of American robins as reservoir hosts for Lyme disease spirochetes.

**DOI:** 10.3201/eid0602.000205

**Published:** 2000

**Authors:** D Richter, A Spielman, N Komar, F R Matuschka

**Affiliations:** Harvard School of Public Health, Boston, MA, USA. dania.richter@charite.de

## Abstract

To explore the competence of American robins as a reservoir for Lyme disease spirochetes, we determined the susceptibility of these birds to tickborne spirochetes and their subsequent infectivity for larval vector ticks. Robins acquired infection and became infectious to almost all xenodiagnostic ticks soon after exposure to infected nymphal ticks. Although infectivity waned after 2 months, the robins remained susceptible to reinfection, became infectious again, and permitted repeated feeding by vector ticks. In addition, spirochetes passaged through birds retained infectivity for mammalian hosts. American robins become as infectious for vector ticks as do reservoir mice, but infectivity in robins wanes more rapidly.


### Competence of American Robins as Reservoir Hosts for Lyme Disease Spirochetes

**Dania Richter,*† Andrew Spielman,* Nicholas Komar,* and Franz-Rainer Matuschka*†**
*Harvard School of Public Health, Boston, Massachusetts, USA; and †Charité, Medizinische Fakultät der Humboldt-Universität zu Berlin, Germany

To explore the competence of American robins as a reservoir for Lyme disease spirochetes, we determined the susceptibility of these birds to tickborne spirochetes and their subsequent infectivity for larval vector ticks. Robins acquired infection and became infectious to almost all xenodiagnostic ticks soon after exposure to infected nymphal ticks. Although infectivity waned after 2 months, the robins remained susceptible to reinfection, became infectious again, and permitted repeated feeding by vector ticks. In addition, spirochetes passaged through birds retained infectivity for mammalian hosts. American robins become as infectious for vector ticks as do reservoir mice, but infectivity in robins wanes more rapidly.

Lyme disease spirochetes (*Borrelia burgdorferi* s.l.) perpetuate in cycles involving rodent reservoir hosts, such as white-footed mice (*Peromyscus leucopus*) in eastern North America (1) and *Apodemus* spp. mice in Eurasia (2). Such hosts readily become infectious to vector ticks and appear to remain so lifelong. They serve as natural reservoirs of infection because numerous subadult vector ticks parasitize them, they are abundant, and they remain in constant residence in tick-infested sites. Rats (*Rattus norvegicus* and *R. rattus*) (3,4) and European dormice (*Glis glis* and *Eliomys quercinus*) (5,6) are similarly competent and locally important hosts to these pathogens. Although hares (*Lepidus timidus*) support infection with Lyme disease spirochetes in Europe (7), the competence of rabbits for these spirochetes has not been proven. A related spirochete, *B. andersonii*, appears to be specific to American rabbits (8,9). Ungulates, however, are not competent as hosts for Lyme disease spirochetes. Deer in intensely enzootic sites fail to infect vector ticks (10,11), and cattle and sheep exposed to infected vector ticks never become infectious (12). Lyme disease spirochetes infect diverse mammals, but not all of them serve as competent hosts.

Certain birds may also contribute to the transmission of Lyme disease spirochetes. Numerous vector ticks parasitize birds in nature, including spirochete-infected larval ticks that presumably acquired infection from these birds (13-19). Seabirds appear to maintain such a cycle on Arctic islands (20). In an enzootic site, American robins (*Turdus migratorius*) are considered to be an avian candidate as a reservoir of infection because they are locally abundant, forage in ground and brush vegetation, and are frequently infested by vector ticks (21). Catbirds (*Dumetella carolinensis*) in enzootic sites, however, appear not to infect vector ticks (22). Although spirochetes have been isolated from naturally infected European blackbirds (*Turdus merula*) (15), a laboratory study failed to demonstrate reservoir competence of these birds (23); the reason for this discrepancy remains unclear. Nymphal vector ticks occasionally become infected after feeding on spirochete-exposed pheasants (*Phasianus colchicus*); these birds, however, cannot contribute to transmission because larval ticks seem not to feed on them, either in the laboratory or in nature (24,25).

Address for correspondence: Dania Richter, Institut für Pathologie, Charité, Medizinische Fakultät der Humboldt-Universität zu Berlin, Malteserstraße 74-100, 12249 Berlin, Germany; fax: 49-30-776-2085; e-mail: dania.richter@charite.de.

Chickens (*Gallus gallus*) become infectious, but only transiently and only when they are about a week old (26). Spirochetes inoculated by syringe can be detected in various tissues of canaries (*Serinus canaria*), bobwhite quail (*Colinus virginianus*), and Japanese quail (*Coturnix coturnix*) (27-29). The competence of candidate reservoir birds as hosts for tickborne spirochetes has not been thoroughly evaluated.

Tickborne Lyme disease spirochetes may readily infect certain birds, and these birds may subsequently infect numerous vector ticks. Therefore, we determined the susceptibility of American robins to tickborne spirochetes and their subsequent infectivity for larval deer ticks (*Ixodes dammini*, which differ from *I. scapularis* [30]). American robins are suitable candidate hosts because they are infested naturally by numerous vector ticks. We evaluated the reservoir competence of captive robins and determined whether they could subsequently be reinfested and reinfected by vector ticks and whether spirochetes passaged through birds remain infective to rodents.

### Materials and Methods

American robins were captured in mist nets in Brookline, MA (U.S. Fish and Wildlife Scientific Collecting Permit# MB719506-0), and maintained at 20°C with a photoperiod of 16:8 hrs (L:D) (Animal Welfare Assurance# A-3431-01). To establish that these birds had not already been infected by Lyme disease spirochetes, nymphs derived from xenodiagnostic larval deer ticks that had engorged on them were examined by dark-field microscopy. No spirochete-infected ticks were found. The birds were held in captivity for 6 months before they were exposed to infected ticks.

The colony of laboratory-raised deer ticks was originally isolated from ticks captured in Ipswich, MA, and was maintained by feeding adults on rabbits and subadults on outbred laboratory mice. To infect ticks, larvae were fed on outbred laboratory mice infected with the N40 strain of *B. burgdorferi* s.s. The resulting infected nymphal ticks were used to infect the robins. Xenodiagnosis was used to determine whether host animals had become infected by spirochetes; larval ticks were permitted to feed on them and the resulting nymphs were examined for spirochetes by dark-field microscopy. The larval ticks used for the xenodiagnosis were in their third generation of continuous laboratory breeding and had not previously been exposed to spirochete-infected hosts. Engorged ticks were held at 20±2°C over supersaturated $MgSO_4$ in sealed desiccator jars with 16 hours of light per day. Hatched or molted ticks remained in screened vials until they were placed on hosts 2-4 months later.

To infest the robins with subadult ticks, each bird was restrained while ticks were brushed onto its head and neck. Each bird was then placed in a cotton bag suspended over water and kept in the dark for approximately 3 hours to limit their activity. The birds were then caged individually over pans of water in cages with wire-mesh floors and fronts (Safeguard Products, New Holland, PA). The contents of the pans were inspected twice a day, and detached ticks were removed promptly. The birds were exposed three times to nymphal ticks to test their susceptibility to repeated feeding (Figure 1). The first and last of these infestations also served to infect them. Xenodiagnostic larval ticks were permitted to feed on these robins nine times during the study.

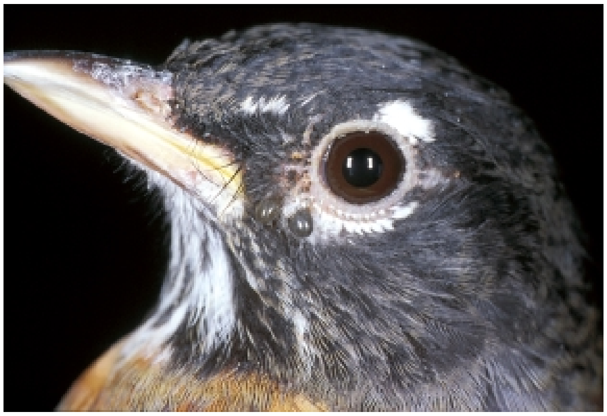

Figure 1. American robins with attached nymphal deer ticks (*Ixodes dammini*).

### Results

To evaluate the reservoir competence of birds for Lyme disease spirochetes, we exposed each of four American robins to the bites of 12 spirochete-infected nymphal deer ticks and determined the duration of their subsequent infectivity for larval deer ticks. The presence of spirochetes in these infecting nymphs was verified by dark-field microscopic examination of the gut contents of the resulting adults; virtually all (97%) contained spirochetes. Birds were exposed initially to xenodiagnostic larvae at the same time they were exposed to the infecting nymphs. Xenodiagnosis was repeated after two days and at intervals for six months. Although larval ticks feeding simultaneously with the infected nymphs failed to acquire spirochetes, two birds infected xenodiagnostic larvae within 6 days after exposure to infected nymphs (Figure 2). All four birds were infectious to xenodiagnostic ticks that

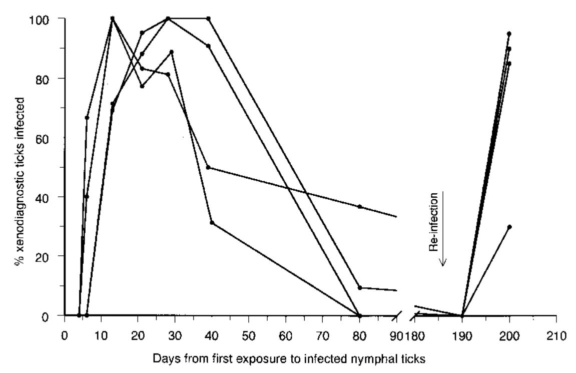

Figure 2. Infectivity to larval vector ticks of four American robins exposed to nymphal deer ticks infected with Lyme disease spirochetes on days 0 and 186. Each observation was recorded on the day on which each xenodiagnostic test was complete.

detached 12 days after exposure and infected 88% (±6.6) of ticks for at least another 3 weeks (Figure). Infectivity waned by 2 months and disappeared by 6 months. Almost all the ticks became infected that fed 2 and 4 weeks after the birds were infected (Table 1). American robins are fully but transiently competent as hosts for Lyme disease spirochetes.

We then determined whether American robins tolerate reinfection by tickborne spirochetes. Six months after they were initially infected, when vector ticks no longer acquired spirochetes from them, eight infected nymphs were permitted to engorge on each bird. All four robins regained infectivity for ticks within the next 2 weeks (Figure 2). Virtually all xenodiagnostic larval ticks that fed on three of these reinfected birds acquired spirochetes, and a third of them did so on the fourth bird. Although spirochete-infected robins only transiently infect vector ticks, the birds remain tolerant to reinfection and become infectious again.

Table 1. Infectivity to larval deer ticks of four American robins infected with tickborne Lyme disease spirochetes

| Days from exposure until xenodiagnosis[a] | Xenodiagnostic ticks No. examined | % infected | ± SE[b] |
|---|---|---|---|
| 0-5 | 16 | 0 | 0 |
| 6-9 | 28 | 28.6 | 16.5 |
| 12-15 | 52 | 84.6 | 7.0 |
| 20-23 | 78 | 85.9 | 6.0 |
| 28-30 | 62 | 91.9 | 6.7 |
| 39-41 | 50 | 68.0 | 20.3 |
| 79-82 | 65 | 13.8 | 10.2 |
| 189-191[c] | 32 | 0 | 0 |
| 199-201 | 80 | 75.0 | 15.2 |

[a]Each interval indicates the days on which xenodiagnostic ticks detached from their hosts.
[b]SE = standard error
[c]Birds were exposed again to infected nymphal ticks on day 186.

The ability of nymphal deer ticks to reinfest American robins was evaluated by recording their feeding success. On initial exposure, 96% of 48 nymphs feeding on the four birds engorged successfully; 98% of 40 nymphs engorged 3 weeks later; and 82% of 32 nymphs engorged 6 months later, when the birds were exposed for the third time. The robins appear to freely tolerate repeated feeding by nymphal deer ticks.

To determine whether spirochetes passaged through birds retain infectivity for mice, we permitted nymphs that had acquired infection as larvae from birds to feed on mice. The ticks that were used had acquired spirochetes 3 weeks after their avian hosts had been infected. Each of four outbred laboratory mice was exposed to six of the resulting nymphs. Two weeks later, 10 xenodiagnostic larval ticks were permitted to engorge on each of these mice. All but one of the 40 resulting nymphal ticks acquired spirochetes (Table 2). Spirochetes, therefore, remain infectious to mammals after a tickborne passage through birds.

### Discussion

A competent reservoir host for the agent of Lyme disease readily acquires infection from vector ticks, permits spirochetes to proliferate, and readily infects vector ticks. In white-footed mice, infection generally becomes established after a single feeding by an infectious tick, and more than half retain infection for approximately 6 months (31). As many as three-quarters of the

Table 2. Infectivity for laboratory mice of Lyme disease spirochetes that had previously infected American robins

| | Avian host | | | | | Murine host | | | |
|---|---|---|---|---|---|---|---|---|---|
| | Infecting nymphs | | Xenodiagnostic ticks[a] | | | Infecting nymphs | | Xenodiagnostic ticks | |
| Bird no. | No. ticks | % engorged | No. examined | % infected | Mouse no. | No. ticks | % engorged | No. examined | % infected |
| 1 | 12 | 83.3 | 18 | 83.3 | 1 | 6 | 100 | 10 | 90 |
| 2 | 12 | 100 | 21 | 95.2 | 2 | 6 | 83.3 | 10 | 100 |
| 3 | 12 | 100 | 22 | 77.3 | 3 | 6 | 100 | 10 | 100 |
| 4 | 12 | 100 | 17 | 88.2 | 4 | 6 | 83.3 | 10 | 100 |

[a]Xenodiagnostic ticks that had engorged on each bird were used to infect the corresponding mouse.

larval ticks that feed on such mice acquire infection, which subsequently wanes. Hamsters are similarly infectious (32). The various murids, glirids, voles, and sciurids that have been tested appear as reservoir-competent for spirochetes as are cricetids (2,5,6,33). Although certain genospecies of the Lyme disease spirochetes are said to be more mouse-adapted than others (34), no experimental evidence is available to support this concept. Rodents, in general, are readily infected by Lyme disease spirochetes, and most remain infectious to vector ticks for at least 6 months.

The standard of proof that has been applied to rodent reservoirs of spirochetal infection has not previously been applied to candidate avian reservoirs. Certain birds, including quail and canaries, can readily be infected by syringe-inoculated spirochetes. Spirochetal DNA generally becomes detectable in the viscera (27-29), and viable spirochetes can be cultured from these tissues. Tickborne spirochetes infect pheasants (24) and week-old chickens (26). About a third of these chicks infect ticks, but they remain infectious for only a week. Adult pheasants infect about a quarter of vector ticks that engorge on them. European blackbirds appear not to be susceptible to tickborne infection (23). American robins, however, appear far more reservoir-competent. Robins bitten by spirochete-infected nymphal ticks subsequently infect larval ticks, and virtually all larvae become infected after feeding on these birds throughout the following month. Infectivity declines, however, and few ticks acquire infection from these birds after approximately 3 months. Certain, but not all, bird species can readily be infected by Lyme disease spirochetes and subsequently become highly but transiently infectious.

A competent reservoir host tolerates feeding by both subadult stages of vector ticks. Although nymphs feed readily on pheasants, larvae seldom attach to these birds, either in nature or in the laboratory (24). About as many nymphal as larval deer ticks attach naturally to American robins (21). Several times as many larvae as nymphs, however, feed on white-footed mice in nature (35). Nymphal vector ticks attach more readily to rats, dormice, or the American reservoir host (*P. leucopus*) than to European mice (*Apodemus* spp.) or voles (2,3,5,31). Perhaps particular subadult stages of vector ticks are more readily attracted to certain vertebrate animals than to others.

To be competent as reservoir hosts for Lyme disease spirochetes, an animal must tolerate repeated feedings by vector ticks. White-footed or laboratory mice serve as hosts to deer ticks almost as readily during their fifth exposure as their first (36). Voles are much less tolerant (37), and rabbits (unpub. obs.) and guinea pigs (38) seldom tolerate more than one episode of attachment. Vector ticks feed repeatedly on American robins as successfully as on the natural rodent reservoirs of the Lyme disease spirochete.

Spirochete-infected larval ticks frequently are taken from birds in Lyme disease-enzootic areas. Of 20 species of North American passerine birds that were parasitized by larval vector ticks, 45% hosted spirochete-infected larvae (13,18). Spirochetes infected 10% to 40% of these batches of infected ticks. In Europe, 81% of 16 bird species harbored infected ticks; of these cohorts, 11% to 75% of the larval ticks were infected (17). Because Lyme disease spirochetes infect <1% of questing larval vector ticks captured in nature and because inherited infection is exceedingly rare (39,40), these larval ticks most likely acquired spirochetes from their avian hosts. Nevertheless, the avian host-range of Lyme disease spirochetes remains to be defined.

An effective reservoir of infection for a pathogen would generate for each primary infection at least as many secondary infections. The contribution of various alternative reservoir hosts for Lyme disease spirochetes, however, can be compared by estimating the proportion of infections in nymphal vector ticks that derive from a particular vertebrate population. This calculation includes estimates of feeding density of vector ticks on the candidate reservoir host, its local density, and its prevalence of infection (2,5). At least twice as many vector ticks appear to acquire infection from a population of edible dormice (*Glis glis*) as from other rodents locally abundant in Central Europe (5). Similar evidence implicates white-footed mice in eastern North America (1). Information that would permit comparable estimates of the reservoir significance of local populations of birds, however, remains unavailable. Thus, it is still uncertain whether a bird, although reservoir-competent for the spirochete, may serve as an important reservoir in nature.

Because the prevalence of Lyme disease spirochetes in American robins has not yet been determined for a representative enzootic site, the importance of these birds as reservoir hosts remains speculative. Robins may contribute to the force of transmission of the agent of Lyme disease, because these locally abundant birds are reservoir-competent and may be infested by numerous larval ticks. Of the larvae that feed on peridomestic birds in North American enzootic sites, approximately three-quarters appear to engorge on robins (21). Although they may be about as abundant as white-footed mice, robins appear to forage more frequently on lawns than do mice (21). The contribution of robins to the peridomestic risk for Lyme disease may depend on the ability of engorged larval ticks detaching from the birds to develop in such habitat. Questing nymphal ticks appear less prevalent on lawns than on other vegetation in residential enzootic sites (41). Birds are more vagile than mice, which might dilute risk by diffusing infected ticks into sites in which transmission is unlikely. Alternatively, their vagility might seed new foci of transmission, if ticks detach in sites that are suitable for their development. Certain migratory passerines have been associated with long-distance dispersal of vector ticks (17-19). Robins may contribute to the emergence of Lyme disease in previously unaffected sites to the extent that the season of their migration overlaps with that of the activity of subadult vector ticks. Our finding that American robins are reservoir-competent for Lyme disease spirochetes warrants further epizootiologic studies, including estimates of the prevalence of spirochetes in these birds in nature.

This study was supported by grant Ma 942/10-1 from the Deutsche Forschungsgemeinschaft and by grant AI 42402-01 from the National Institutes of Health.

Dr. Richter is a postdoctoral fellow conducting joint research in the laboratories of Dr. Matuschka at the Charité Medical School, Humboldt-Universität zu Berlin, and Dr. Spielman, at the Harvard School of Public Health. Her research interests focus on the immunologic and molecular interface of the host-vector-pathogen relationship in the epizootiology of Lyme disease.

### References

1. Spielman A. Lyme disease and human babesiosis: evidence incriminating vector and reservoir hosts. In: Englund PT, Sher A, editors. The biology of parasitism. New York: Alan R. Liss; 1988. p. 147-65.
2. Matuschka F-R, Fischer P, Heiler M, Richter D, Spielman A. Capacity of European animals as reservoir hosts for the Lyme disease spirochete. J Infect Dis 1992;165:479-83.
3. Matuschka F-R, Endepols S, Richter D, Ohlenbusch A, Eiffert H, Spielman A. Risk of urban Lyme disease enhanced by the presence of rats. J Infect Dis 1996;174:1108-11.
4. Smith RP, Rand PW, Lacombe EH, Telford SR, Rich SM, Piesman J, et al. Norway rats as reservoir hosts for Lyme disease spirochetes on Monhegan Island, Maine. J Infect Dis 1993;168:687-91.
5. Matuschka F-R, Eiffert H, Ohlenbusch A, Spielman A. Amplifying role of edible dormice in Lyme disease transmission in central Europe. J Infect Dis 1994;170:122-7.
6. Matuschka F-R, Allgöwer R, Spielman A, Richter D. Characteristics of garden dormice that contribute to their capacity as reservoirs for Lyme disease spirochetes. Appl Environ Microbiol 1999;65:707-11.
7. Jaensen TGT, Tälleklint L. Lyme borreliosis spirochetes in *Ixodes ricinus* (Acari: Ixodidae) and the varying hare on isolated islands in the Baltic Sea. J Med Entomol 1996;33:339-43.
8. Anderson JF, Magnarelli LA, LeFebvre RB, Andreadis TG, McAninch JB, Perng G-C, et al. Antigenically variable *Borrelia burgdorferi* isolated from cottontail rabbits and *Ixodes dentatus* in rural and urban areas. J Clin Microbiol 1989;27:13-20.
9. Marconi RT, Liveris D, Schwartz I. Identification of novel insertion elements, restriction fragment length polymorphism patterns, and discontinuous 23S rRNA in Lyme disease spirochetes—phylogenetic analyses of rRNA genes and their intergenic spacers in *Borrelia japonica* sp. nov. and genomic group 21038 (*Borrelia andersonii* sp. nov.) isolates. J Clin Microbiol 1995;33:2427-34.

10. Matuschka F-R, Heiler M, Eiffert H, Fischer P, Lotter H, Spielman A. Diversionary role of hooded game in the transmission of Lyme disease spirochetes. Am J Trop Med Hyg 1993;48:693-9.
11. Telford SR, Mather TN, Moore SI, Wilson ML, Spielman A. Incompetence of deer as reservoirs of the Lyme disease spirochete. Am J Trop Med Hyg 1988;39:105-9.
12. Matuschka F-R, Eiffert H, Ohlenbusch A, Richter D, Schein E, Spielman A. Transmission of the agent of Lyme disease on a subtropical island. Tropical Medicine and Parasitology 1994;45:39-44.
13. Anderson JF, Johnson RC, Magnarelli LA, Hyde FW. Involvement of birds in the epidemiology of the Lyme disease agent *Borrelia burgdorferi*. Infect Immun 1986;51:394-6.
14. Anderson JF, Magnarelli LA, Stafford KC. Bird-feeding ticks transstadially transmit *Borrelia burgdorferi* that infect Syrian hamsters. J Wildl Dis 1990;26:1-10.
15. Humair P-F, Postic D, Wallich R, Gern L. An avian reservoir (*Turdus merula*) of the Lyme borreliosis spirochetes. Zentralbl Bakteriol 1998;287:521-38.
16. Nakao M, Miyamoto K, Fukunaga M. Lyme disease spirochetes in Japan: enzootic transmission cycles in birds, rodents, and *Ixodes persulcatus* ticks. J Infect Dis 1994;170:878-82.
17. Olsen B, Jaenson TGT, Bergström S. Prevalence of *Borrelia burgdorferi* sensu lato-infected ticks on migrating birds. Appl Environ Microbiol 1995;61:3082-7.
18. Rand PW, Lacombe EH, Smith RP, Ficker J. Participation of birds in the emergence of Lyme disease in southern Maine. J Med Entomol 1998;35:270-6.
19. Smith RP, Rand PW, Lacombe EH, Morris SR, Holmes DW, Caporale DA. Role of bird migration in the long-distance dispersal of *Ixodes dammini*, the vector of Lyme disease. J Infect Dis 1996;174:221-4.
20. Olsen B, Jaenson TGT, Noppa L, Bunikis J, Bergström S. A Lyme borreliosis cycle in seabirds and *Ixodes uriae* ticks. Nature 1993;362:340-2.
21. Battaly GR, Fish D. Relative importance of bird species as hosts for immature *Ixodes dammini* (Acari: Ixodidae) in a suburban residential landscape of southern New York State. J Med Entomol 1993;30:740-7.
22. Mather TN, Telford SR, MacLachlan AB, Spielman A. Incompetence of catbirds as reservoirs for the Lyme disease spirochete (*Borrelia burgdorferi*). J Parasitol 1989;75:66-9.
23. Matuschka F-R, Spielman A. Loss of Lyme disease spirochetes from *Ixodes ricinus* ticks feeding on European blackbirds. Exp Parasitol 1992;74:151-8.
24. Kurtenbach K, Carey D, Hoodless AN, Nuttall PA, Randolph SE. Competence of pheasants as reservoirs for Lyme disease spirochetes. J Med Entomol 1998;35:77-81.
25. Kurtenbach K, Peacey M, Rijpkema SGT, Hoodless AN, Nuttall PA, Randolph SE. Differential transmission of the genospecies of *Borrelia burgdorferi* sensu lato by game birds and small rodents in England. Appl Environ Microbiol 1998;64:1169-74.
26. Piesman J, Dolan MC, Schriefer ME, Burkot TR. Ability of experimentally infected chickens to infect ticks with the Lyme disease spirochete, *Borrelia burgdorferi*. Am J Trop Med Hyg 1996;54:294-8.
27. Bishop KL, Khan MI, Nielsen SW. Experimental infection of northern bobwhite quail with *Borrelia burgdorferi*. J Wildl Dis 1994;30:506-13.
28. Isogai E, Tanaka S, Braga IS, Itakura C, Isogai H, Kimura K, et al. Experimental *Borrelia garinii* infection of Japanese quail. Infect Immun 1994;62:3580-2.
29. Olsen B, Gylfe A, Bergström S. Canary finches (*Serinus canaria*) as an avian infection model for Lyme borreliosis. Microbial Pathogenesis 1996;20:319-24.
30. Telford SR. The name *Ixodes dammini* epidemiologically justified. Emerg Infect Dis 1998;4:132-4.
31. Donahue JG, Piesman J, Spielman A. Reservoir competence of white-footed mice for Lyme disease spirochetes. Am J Trop Med Hyg 1987;36:92-6.
32. Piesman J. Intensity and duration of *Borrelia burgdorferi* and *Babesia microti* infectivity in rodent hosts. Int J Parasitol 1988;18:687-9.
33. Matuschka F-R, Endepols S, Richter D, Spielman A. Competence of urban rats as reservoir hosts for Lyme disease spirochetes. J Med Entomol 1997;34:489-93.
34. Humair P-I, Peter O, Wallich R, Gern L. Strain variation of Lyme disease spirochetes isolated from *Ixodes ricinus* ticks and rodents collected in two epidemic areas in Switzerland. J Med Entomol 1995;32:433-8.
35. Wilson ML, Spielman A. Seasonal activity of immature *Ixodes dammini* (Acari: Ixodidae). J Med Entomol 1985;22:408-14.
36. Richter D, Spielman A, Matuschka F-R. Effect of prior exposure to noninfected ticks on susceptibility of mice to Lyme disease spirochetes. Appl Environ Microbiol 1998;64:4596-9.
37. Davidar P, Wilson M, Ribeiro JMC. Differential distribution of immature *Ixodes dammini* (Acari: Ixodidae) on rodent hosts. J Parasitol 1989;75:898-904.
38. Nazario S, Das S, de Silva AM, Deponte K, Marcantonio N, Anderson JF, et al. Prevention of *Borrelia burgdorferi* transmission in guinea pigs by tick immunity. Am J Trop Med Hyg 1998;58:780-5.
39. Matuschka F-R, Schinkel TW, Klug B, Spielman A, Richter D. Failure of *Ixodes* ticks to inherit *Borrelia afzelii* infection. Appl Environ Microbiol 1998;64:3089-91.
40. Piesman J, Donahue JG, Mather TN, Spielman A. Transovarially acquired Lyme disease spirochetes (*Borrelia burgdorferi*) in field-collected larval *Ixodes dammini* (Acari: Ixodidae). J Med Entomol 1986;23:219.
41. Frank DH, Fish D, Moy FH. Landscape features associated with Lyme disease risk in a suburban residential environment. Landscape Ecol 1998;13:27-36.
